# Prevalence, main diagnoses, and outcomes of hospital admissions in Mongolian children: a national registry-based descriptive analysis

**DOI:** 10.3389/fped.2026.1732305

**Published:** 2026-02-10

**Authors:** Altanchimeg Sainbayar, Naranpurev Mendsaikhan, Romana Erblich, Ganbold Lundeg, Myagmarsuren Baterdene, Jens Meier, Martin W. Dünser

**Affiliations:** 1 Department of Critical Care and Anesthesia, Mongolian National University of Medical Sciences, Ulaanbaatar, Mongolia; 2 Intensive Care Unit, Mongolia Japan Hospital, Ulaanbaatar, Mongolia; 3 Department of Anesthesiology and Intensive Care Medicine, Kepler University Hospital and Johannes Kepler University, Linz, Austria; 4 Department of Pediatrics, Songinokhairkhan General Hospital, Ulaanbaatar, Mongolia

**Keywords:** causes of death, hospital admission, hospitalization, Mongolia, newborns, under-five children

## Abstract

**Background:**

No systematic data on pediatric hospital admissions in Mongolia are available.

**Methods:**

The Mongolian National Hospital Data registry was screened for pediatric (<18 years) hospital admissions (01.01.2019–31.12.2023) to report the prevalence, main diagnoses, and outcomes of hospital admissions in Mongolian newborns, post-neonatal children und 5 years, and children aged 5–17.99 years. Descriptive methods were used for data analysis.

**Results:**

During the study period, 927,223 pediatric hospital admissions were identified translating into a median prevalence of 5,218 (IQR: 4,386–6,098) pediatric hospital admissions per 100,000 population (all ages) per year. This prevalence was highest among post-neonatal children under 5 years (median: 3,780; IQR: 2,491–3,900), followed by children aged 5–17.99 years (median: 1,945; IQR: 1,929–2,071), and newborns (median: 275; IQR: 246–295) over the study period. Neonatal jaundice was the most frequent main diagnosis in newborns. Pneumonia and COVID-19 were most common in both post-neonatal children under 5 years and children aged 5–17.99 years. The median length of hospital stay was 6.7 (IQR: 5.0–9.0) days in newborns, 6.0 (IQR: 5.0–7.0) days in post-neonatal children under 5 years, and 7.0 (IQR: 5.0–8.0) days in children aged 5–17.99 years. The hospital mortality rate was 5.9% in newborns, 0.25% in post-neonatal children under 5 years, and 0.14% in children aged 5–17.99 years.

**Conclusions:**

This nationwide, registry-based study found a median prevalence of 5,218 pediatric hospital admissions per 100,000 population (all ages) per year in Mongolia. The majority of pediatric hospital admissions occurred in post-neonatal children under five years. The observation period included the COVID-19 pandemic years.

## Introduction

Mongolia is a lower-middle income country in Central Asia and with two people per square-kilometer the least densely populated in the world (1). In 2023, the median age of the Mongolian population was 27 years with one in six Mongolians younger than fifteen years (1, 2). Since the transition from Soviet influence to a democratic, market economy, Mongolia has experienced a dramatic reduction in its neonatal, infant and under-five mortality reaching a nadir of 7.4, 12.0 and 13.6 deaths per 1,000 live births in 2023, respectively (3). A situation analysis of “Well-Child Care in Mongolia” by the World Health Organization reported that the leading causes of death among children under the age of five years had shifted from communicable to non-communicable diseases with congenital anomalies and injuries being most common (4). In 2021, two thirds of all deaths in children younger than five years were reported to have occurred from preventable diseases (5).

The Mongolian hospital system is organized in three tiers including primary level facilities, secondary and tertiary hospitals (6). Hospital-based pediatric services are almost exclusively offered in secondary province hospitals as well as dedicated pediatric tertiary centers in the capital city of Ulaanbaatar. At the sub-province or *Soum* level, the healthcare needs of children are covered by primary care services. While data on the prevalence, cause and outcomes of hospital admissions in Mongolian adults have recently been published (7), no information on these indicators in children exists. Availability of such data could help to inform the future planning and financing of hospital-based pediatric services in the country.

The aim of this study was to report the prevalence, main diagnoses, and outcomes of pediatric hospital admissions in Mongolian newborns, post-neonatal children under five years, as well as children aged 5–17.99 years from January 1, 2019 until December 31, 2023.

## Materials and methods

This study was designed as a descriptive analysis of a national registry. It included data from all pediatric inpatient admissions to public and private hospitals in Mongolia during the time from January 1, 2019 until December 31, 2023. The study was part of a larger research project to determine the prevalence, cause and outcomes of hospital admissions in Mongolia. The study focusing on these outcomes in adult patients has recently been published (7). The research protocol for both analyses (adults and children) was evaluated and approved by the Ethics Committee of the Mongolian National University of Medical Sciences (2021/3–11). In view of the retrospective design written informed consent was waived.

This study was supported by institutional funds by the Mongolian National University of Medical Sciences only. The Mongolian National University of Medical Sciences had no influence on the study design, data collection, data analysis, interpretation of the results, writing of the manuscript or the decision to submit the paper for publication. All authors had full access to the trial data, critically reviewed the manuscript, and vouch for the analysis, accuracy and completeness of the study data. The manuscript was drafted according to the updated STROBE checklist for reporting cohort studies (8).

### The national hospital data registry

The National Hospital Data registry is hosted by the National Statistics Office of Mongolia. This registry aggregates data on all inpatient admissions to public and private hospitals across Mongolia. Using a standardized online data entry tool, patient-related, medical and administrative data of each hospital admission are entered by dedicated physicians trained in the use of the online data collection tool. Variables and data entry into the online registry are standardized and summarized in an instruction manual. All data are aggregated, updated daily, and monitored by the Center for Health Development of Mongolia (9). The latter institution assures completeness of the datasets by retrieving missing data from submitting bodies at regular intervals. Following clearance by the Center for Health Development of Mongolia, data are finally transferred to the National Statistics Office for detailed analysis (10).

### Study population

All patients, who were younger than 18 years and admitted to a Mongolian hospital during the study period, were eligible for study inclusion. Records without patient data, with missing demographic patient data (defined as either unavailable age or sex), as well as records of adult patients (age ≥18 years) were excluded. A hospital was defined as a healthcare institution, which (1) offered diagnostic and therapeutic patient services for medical conditions, (2) had at least six beds, (3) had an organized physician staff, and (4) provided continuous nursing services under the supervision of a registered nurse (11). An inpatient admission was defined as a course of inpatient treatment in a hospital with an intended duration of at least one overnight stay (12). For the purpose of the present study, hospital admissions were counted per admission and not per patient meaning that individual patients with repeated hospital admissions or those transferred from one hospital to another were included into the analysis more than once.

### Study variables and definitions

The following data were extracted from the National Hospital Data registry for all study participants: age, sex, region of residence, date of hospital admission, level of admission hospital, main diagnosis leading to hospital admission as indicated by the International Statistical Classification of Diseases and Related Health Problems (ICD)-10 code (13), length of hospital stay, and survival status at hospital discharge. Based on the age cut-off suggested by the United Nations (14), children were defined as human beings under the age of 18 years. For this study, children were further categorized into the following three age groups: (1) newborns (<28 days); (2) post-neonatal children under five years (28 days to 4.99 years); and (3) children aged 5–17.99 years. To calculate the prevalence of hospital admissions per 100,000 population (all ages) per year, the number of registered inhabitants (children and adults) per province and region in each of the five study years was collected from the National Statistics Office of Mongolia. The regions of residence were grouped into the following six regions of Mongolia: East: Dornod, Sukhbataar, and Khentii province; North: Arkhangai, Bulgan, Orkhon, Khuvsgul, Selenge, and Darkhna-Uul province; Center: Dornogobi, Tuv and Uvurkhangai province; South: Bayankhongor, Umnugobi, Gobisumber, and Dundgobi province; West: Bayan-Ulgii, Gobi-Altai, Zavkhan, Uvs, and Khovd province; Ulaanbaatar: capital city of Ulaanbaatar. Based on the date of hospital admission, the season of admission was determined (winter: December 1–February 28, mean temperature: −17.4 °C; spring: March 1–May 31, mean temperature: 2.7 °C; summer: June 1–August 31, mean temperature: 17.6 °C; autumn: September 1–November 30, mean temperature: 1.1 °C) (15). Admission hospitals were grouped into level 1 (*Soum* hospitals), level 2 (provincial hospitals, district hospitals, private hospitals), and level 3 (university hospitals, specialized hospitals). In order to identify the main diagnosis leading to hospital admission, the ICD-10 code was truncated to the alphabetic character and numeric digit without decimals. The only exception was made for coronarvirus disease 2019 (COVID-19), which was coded with its original full ICD-10 code (J07.1). We grouped all injuries and encoded trauma mechanisms under the main diagnosis of “trauma and injuries”. Similarly, we grouped congenital malformations according to the body regions or organ systems. The survival status at hospital discharge was encoded as either alive or dead. The crude mortality rate was then calculated as the ratio between the number of patients who died during their hospital admission and the total number of hospital admissions.

### Data processing

Study variables were extracted from the National Hospital Data registry for each year and then merged into a single datafile. Plausibility of the single dataset was tested by tabling all variables to identify relevant outliers. In addition, data type checks were performed assuring that non-numeric and numeric variables were not mixed. Wherever possible, data corrections were made. Otherwise, variables were deleted and marked as missing. No data imputation methods were used in case of missing values. Non-numeric variables (*e.g.*, region of residence) were translated into American English using an online available multilingual neural machine translated service (Google Translate®; Google, Mountain View, United States). Using the ICD package for R, ICD-10 codes (truncated to the first decimal, as indicated above) were transformed into main diagnoses.

### Study outcomes

The primary outcome of this study were the absolute number of pediatric hospital admissions and the prevalence of pediatric hospital admissions per 100,000 population (all ages) per year during the study period. The prevalence of pediatric hospital admissions per 100,000 population (all ages) per year was calculated for all study patients as well as for patients stratified into the three age groups (newborns, post-neonatal children under five years, children aged 5–17.99 years). Furthermore, the primary outcome was determined separately for males and females, the seasons of admission, the regions of residence, and levels of admission hospitals across the three age strata. Secondary study outcomes were the ten most common causes of hospital admission as expressed by the ICD-10 coded main diagnoses, the length of hospital stay, as well as the mortality rate at hospital discharge. Secondary outcomes were reported separately for children in the three age groups.

### Statistical analysis

All statistical analyses were performed using the R software package (R version 4.2.3; The R Foundation, Vienna, Austria). Descriptive methods were used to report primary and secondary study outcomes. As normality assumptions (tested by the Kolmogorov–Smirnov test) were not fulfilled for most of the continuous variables, these parameters are presented as median values with interquartile ranges. Categorical variables are presented as absolute numbers with percentages.

## Results

Out of 4,378,364 records in the National Hospital Data registry, 927,223 cases were included into the statistical analysis (Electronic Supplementary Material Figure E1). Less than one percent of all data was missing (Electronic Supplementary Material Figure E2). Table 1 summarizes demographic data, region of residence, season of admission, time periods before, during and after the COVID-19 pandemic and level of the admission hospital in study patients stratified into the three age groups.

**Table 1:** Demographic data, region of residence, season of admission, time periods before, during and after the COVID-19 pandemic, as well as level of the admission hospital in the three age groups.

Variables	Units	Newborns	Under- five children	Children aged 5–17.99 years
	*n*	45,791	550,172	331,260
Age	years	0.03 (0.01–0.05)	1.4 (0.7–2.6)	11.0 (7.7–14.5)
Male sex	*n* (%)	25,921 (56.6%)	304,060 (55.2%)	168,940 (51%)
Region of residence^a^	*n* (%)			
East		2,438 (5.3%)	35,766 (6.5%)	23,133 (7.0%)
North		8,419 (18.4%)	88,319 (16.1%)	65,929 (19.9%)
Center		3,628 (7.9%)	39,555 (7.2%)	29,296 (8.8%)
South		3,662 (8.0%)	31,707 (5.8%)	26,202 (7.9%)
West		4,311 (9.4%)	50,172 (9.1%)	38,430 (11.6%)
Ulaanbaatar		21,409 (46.7%)	281,105 (51.1%)	137,478 (41.5%)
Missing		1,924 (4.2%)	23,548 (4.3%)	10,792 (3.3%)
Season of admission	*n* (%)			
Winter		10,763 (23.5%)	168,438 (30.6%)	76,559 (23.1%)
Spring		12,342 (27.0%)	151,987 (27.6%)	78,552 (23.7%)
Summer		11,349 (24.8%)	93,083 (16.9%)	90,792 (27.4%)
Autumn		11,337 (24.8%)	136,664 (24.8%)	85,357 (25.8%)
COVID-19 pandemic^b^	*n* (%)			
Before (2019–2020)		17,330 (37.8%)	208,949 (38.0%)	120,430 (36.4%)
During (2021–2022)		17,179 (37.5%)	209,650 (38.1%)	138,719 (41.9%)
After (2023)		11,282 (24.6%)	131,573 (23.9%)	72,111 (21.8%)
Level of admission hospital	*n* (%)			
Level 1		2,723 (5.9%)	83,944 (15.3%)	52,058 (15.7%)
Level 2		34,120 (74.5%)	406,074 (73.8%)	213,780 (64.5%)
Level 3		8,948 (19.5%)	60,154 (10.9%)	65,422 (19.7%)

COVID-19, coronavirus disease 2019.

^a^ Regions include the following provinces: East: Dornod, Sukhbataar, and Khentii; North: Arkhangai, Bulgan, Orkhon, Khuvsgul, Selenge, and Darkhna-Uul; Center: Dornogobi, Tuv and Uvurkhangai; South: Bayankhongor, Umnugobi, Gobisumber, and Dundgobi, West: Bayan-Ulgii, Gobi-Altai, Zavkhan, Uvs, and Khovd; Ulaanbaatar: capital city of Ulaanbaatar.

^b^ Using COVID-19 case records of Mongolia as published by the World Health Organization, the five study years were categorized into a time period before (January 1, 2019 until December 31, 2020), during (January 1, 2021 until December 31, 2022), and after the COVID-19 pandemic (January 1 until December 31, 2023).

### Primary study outcome

**Figure 1:**
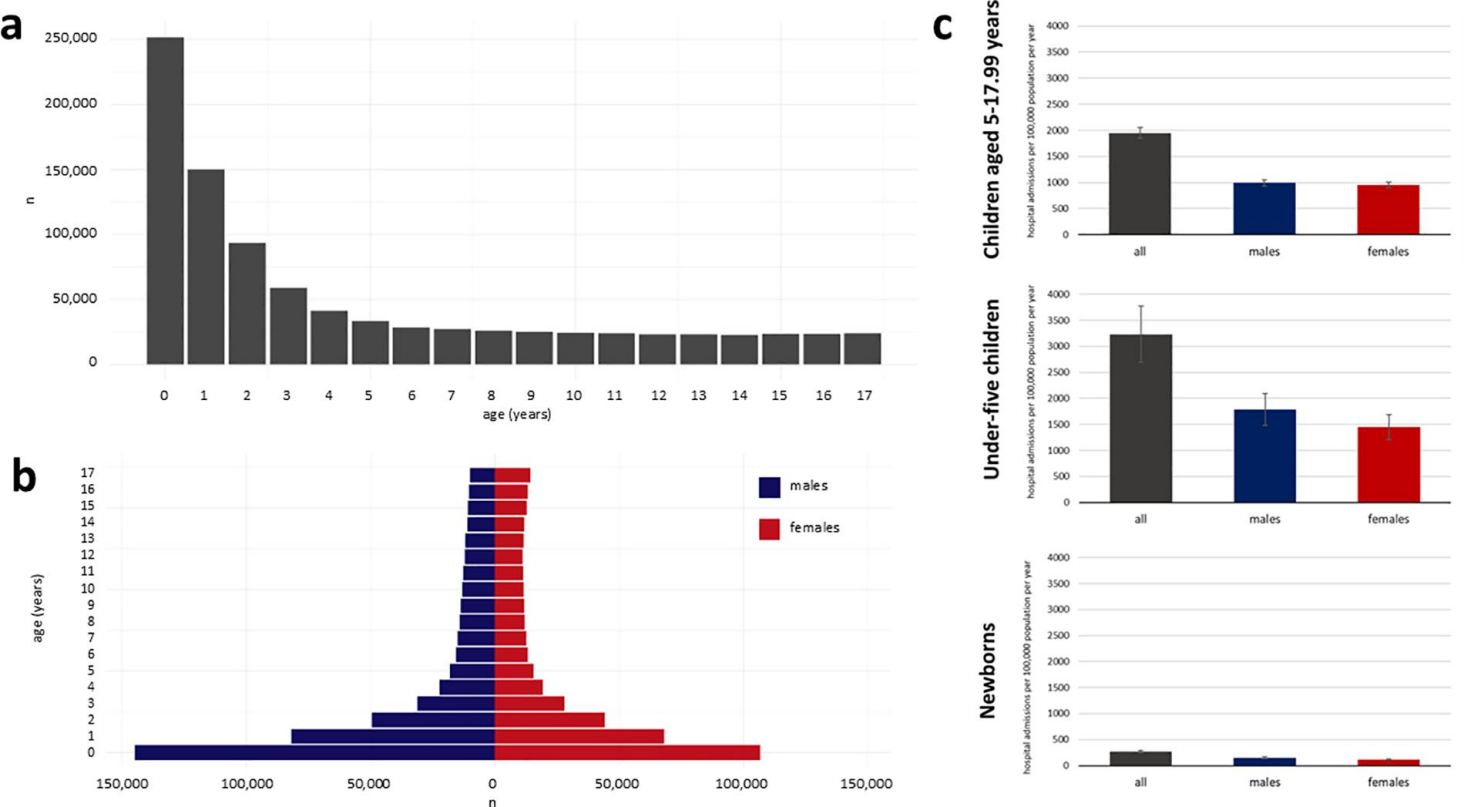
Histogram of pediatric hospital admissions over single years of life **(a)**, age pyramids by sex **(b)** and prevalence of hospital admissions per 100,000 population (all ages) per year for all study patients, males and females in the three age groups **(c)**. yrs, years.

**Figure 2:**
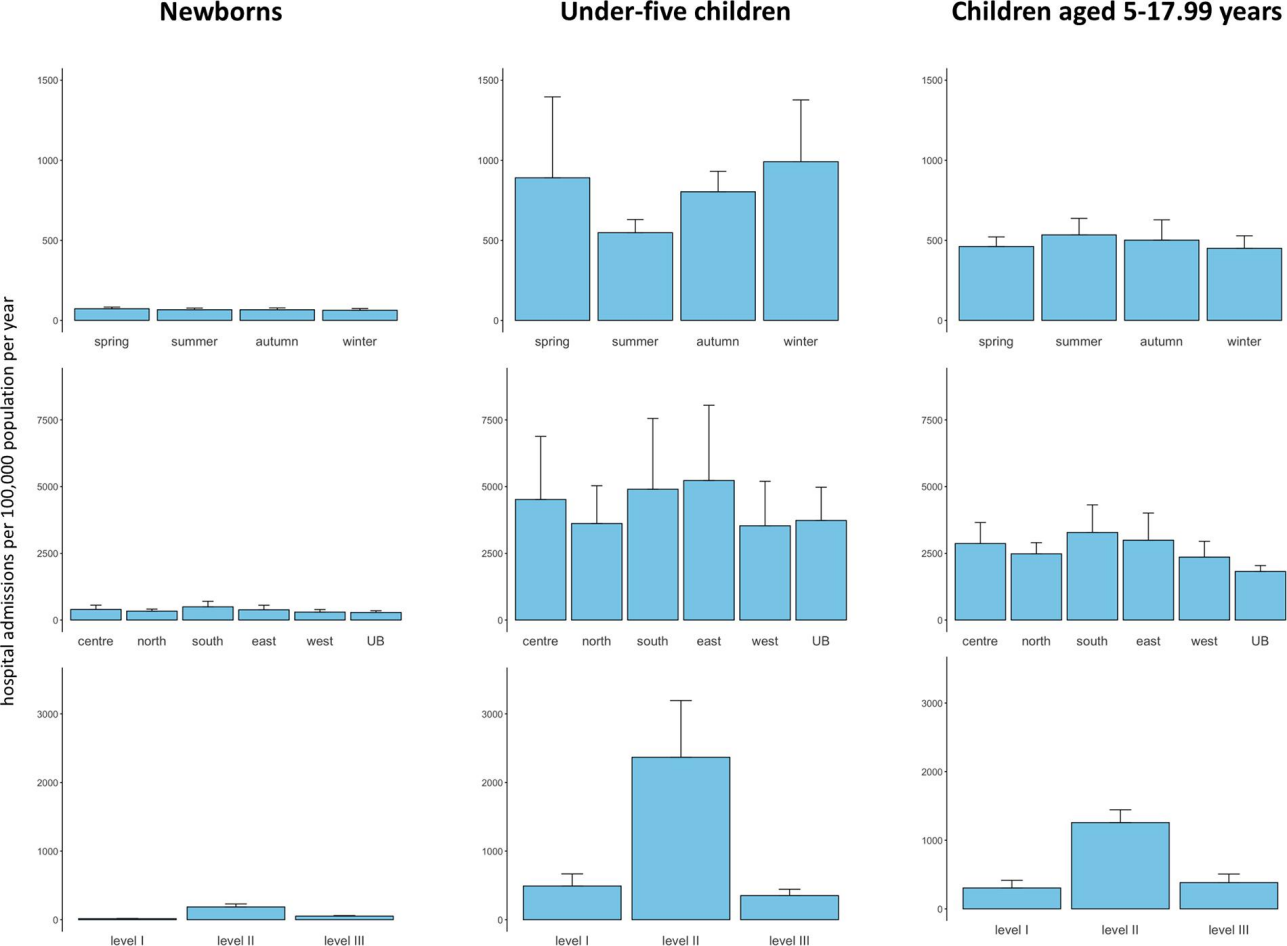
Prevalence of hospital admissions per 100,000 population per year for seasons of admission, regions of residence, and levels of admission hospitals in the three age groups. UB, Ulaanbaatar.

During the five years study period, 927,223 hospital admissions in children were registered. Hospital admissions during the first year, first two years, first three years and first five years of life made up 27.1% (251,5890/927,223), 43.3% (401,550/927,223), 53.4% (495,184/927,223), and 64.3% (595,963/927,223) of all pediatric hospital admissions, respectively (Figure 1a). There was a male predominance with 53.8% of all pediatric hospital admissions including children of male sex (Figure 1b). Over the five years study period, the median prevalence of pediatric hospital admissions per 100,000 population (all ages) per year in Mongolia was 5,218 (IQR, 4,386–6,098). The prevalence of pediatric hospital admissions per 100,000 population (all ages) per year was highest among post-neonatal children under five years followed by children aged 5–17.99 years as well as newborns (Figure 1c). While a seasonal variation in the prevalence of hospital admissions per 100,000 population (all ages) per year was observed in post-neonatal children under five years, this was not seen in the other age groups (Figure 2). The prevalence of pediatric hospital admissions per 100,000 population (all ages) per year was highest among level 2 hospitals across all age groups (Figure 2).

### Secondary study outcomes

**Figure 3:**
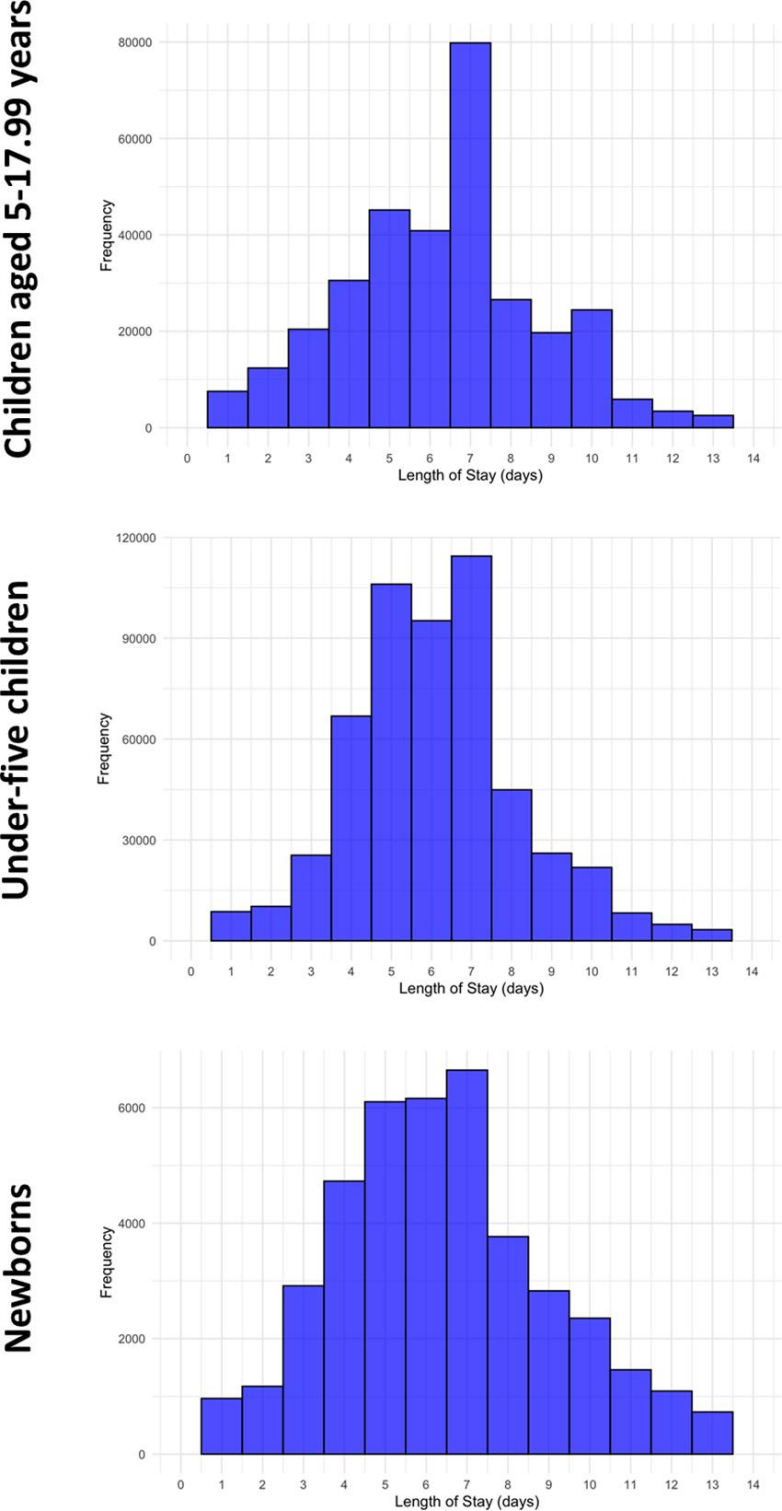
Histograms of hospital lengths of stay in the three age groups (truncated to length of stays <14 days).

**Figure 4:**
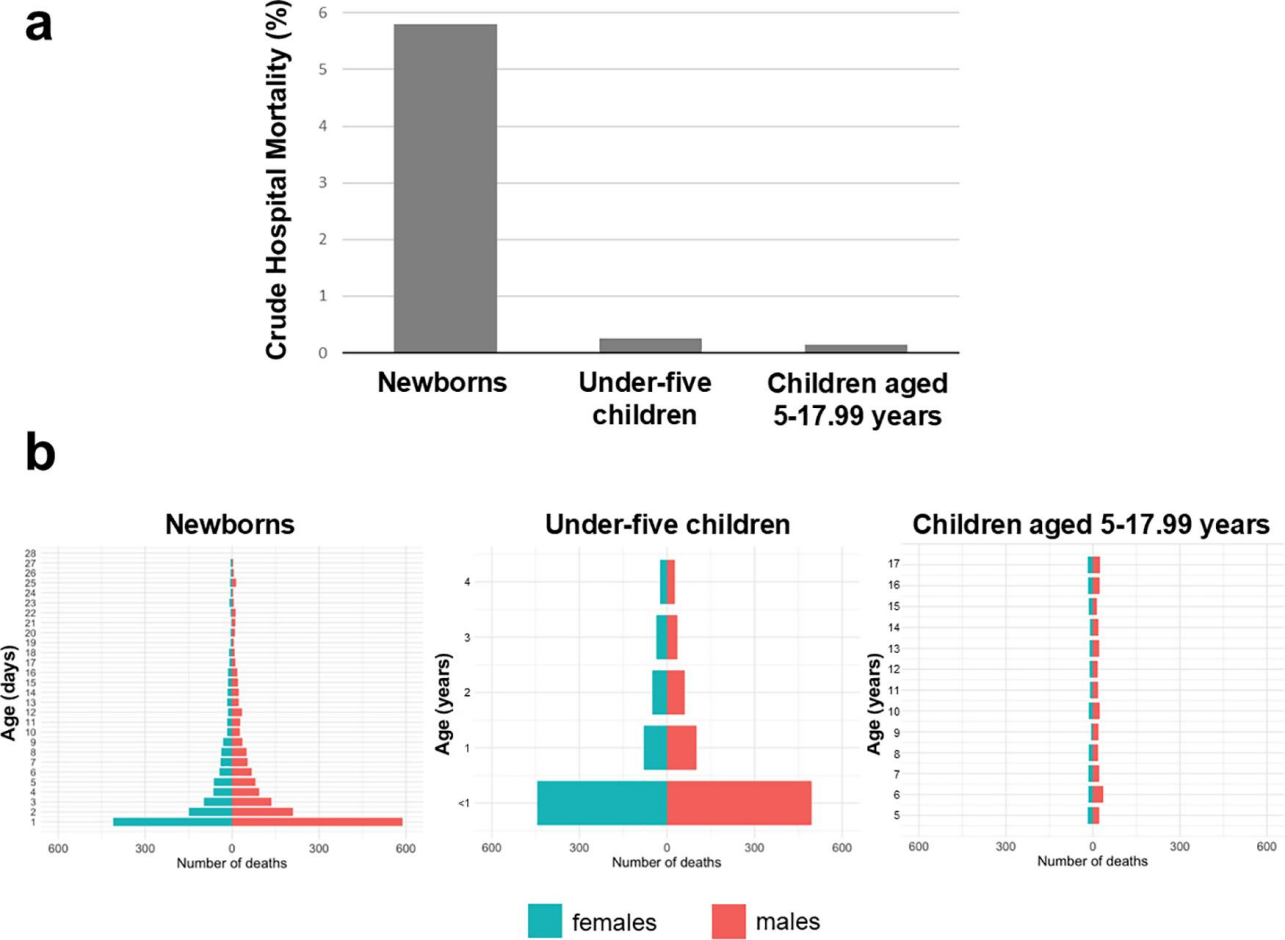
Crude hospital mortality rates **(a)** and age pyramids of deaths by sex **(b)** in the three age groups.

Top-ten main diagnoses leading to pediatric hospital admission in newborns, post-neonatal children under five years, and children aged 5–17.99 years are displayed in Table 2. While neonatal jaundice was the most frequent main diagnosis in newborns (both males and females), pneumonia and COVID-19 were the ones in post-neonatal children under five years and children aged 5–17.99 years irrespective of sex, respectively. The median length of hospital stay was 6.7 (IQR, 5.0–9.0) days in newborns, 6.0 (IQR, 5.0–7.0) days in post-neonatal children under five years, and 7.0 (IQR, 5.0–8.0) days in children aged 5–17.99 years (Figure 3). The all-cause crude hospital mortality rate was 5.9% (2,693/45,791) in newborns, 0.25% (1,353/550,172) in post-neonatal children under five years, and 0.14% (455/331,260) in children aged 5–17.99 years (Figure 4a). The majority of deaths occurred during the first week of life in newborns and during the first year of life in post-neonatal children under five years. No such peak could be observed in children aged 5–17.99 years (Figure 4b). Table 3 reports the crude hospital mortality rates of the ten most common ICD-10-coded (13) main diagnoses, the ICD-10-coded (13) main diagnoses causing most hospital deaths, and the ICD-10 coded (13) main diagnoses with the highest hospital mortality rates for study patients across the three age groups.

**Table 2:** The ten most frequent ICD-10-coded (13) main diagnoses in all study patients, females and males stratified into the three age groups.

Age groups	All			Females			Males		
Newborns (<28 days)	*n* = 45,791			*n* = 19,870			*n* = 25,921		
	1	Neonatal jaundice (P59)	21,646 (47.3%)	1	Neonatal jaundice (P59)	9,092 (45.8%)	1	Neonatal jaundice (P59)	12,554 (48.4%)
	2	Disturbances of cerebral status of newborn (P91)	6,427 (14%)	2	Disturbances of cerebral status of the newborn (P91)	2,857 (14.4%)	2	Disturbances of cerebral status of the newborn (P91)	3,570 (13.8%)
	3	Infections specific to the perinatal period (P39)	1,917 (4.2%)	3	Infections specific to the perinatal period (P39)	868 (4.4%)	3	Infections specific to the perinatal period (P39)	1,049 (4%)
	4	Respiratory distress of the newborn (P22)	1,538 (3.3%)	4	Pneumonia, unspecified organism (J18)	666 (3.4%)	4	Respiratory distress of the newborn (P22)	884 (3.4%)
	5	Pneumonia, unspecified organism (J18)	1,512 (3.3%)	5	Respiratory distress of the newborn (P22)	654 (3.3%)	5	Pneumonia, unspecified organism (J18)	846 (3.3%)
	6	Other respiratory conditions originating in the perinatal period (P28)	1,384 (2.9%)	6	Other respiratory conditions originating in the perinatal period (P28)	627 (3.2%)	6	Other respiratory conditions originating in the perinatal period (P28)	757 (3%)
	7	Congenital pneumonia (P23)	1,064 (2.3%)	7	Congenital pneumonia (P23)	446 (2.2%)	7	Congenital pneumonia (P23)	618 (2.4%)
	8	Omphalitis of newborn (P38)	864 (1.9%)	8	Omphalitis of newborn (P38)	438 (2.2%)	8	Omphalitis of newborn (P38)	426 (1.6%)
	9	Viral pneumonia (non-COVID-19) (J12)	753 (1.6%)	9	Viral pneumonia (non-COVID-19) (J12)	336 (1.7%)	9	Viral pneumonia (non-COVID-19) (J12)	417 (1.6%)
	10	COVID-19 (U07.1)	676 (1.5%)	10	COVID-19 (U07.1)	328 (1.7%)	10	COVID-19 (U07.1)	348 (1.3%)
Under-five children (28 days—4.99) years)	*n* = 550,172			*n* = 246,112			*n* = 304,060		
	1	Pneumonia, unspecified organism (J18)	202,598 (36.8%)	1	Pneumonia, unspecified organism (J18)	91,621 (37.2%)	1	Pneumonia, unspecified organism (J18)	110,911 (36.5%)
	2	Noninfective gastroenteritis and colitis (K52)	61,441 (11.2%)	2	Noninfective gastroenteritis and colitis (K52)	27,232 (11.1%)	2	Noninfective gastroenteritis and colitis (K52)	34,209 (11.3%)
	3	Acute bronchitis (J20)	53,703 (9.8%)	3	Acute bronchitis (J20)	24,348 (9.9%)	3	Acute bronchitis (J20)	29,355 (9.7%)
	4	COVID-19 (U07.1)	29,779 (5.4%)	4	COVID-19 (U07.1)	13,951 (5.7%)	4	Viral pneumonia (non-COVID-19) (J12)	16,054 (5.3%)
	5	Viral pneumonia (non-COVID-19) (J12)	28,921 (5.3%)	5	Viral pneumonia (non-COVID-19) (J12)	12,867 (5.2%)	5	COVID-19 (U07.1)	15,828 (5.2%)
	6	Acute tonsillitis (J03)	16,140 (2.9%)	6	Acute tonsillitis (J03)	7,098 (2.9%)	6	Acute laryngitis and tracheitis (J04)	9,615 (3.2%)
	7	Acute laryngitis and tracheitis (J04)	15,666 (2.8%)	7	Acute laryngitis and tracheitis (J04)	6,051 (2.5%)	7	Acute tonsillitis (J03)	9,042 (3%)
	8	Acute bronchiolitis (J21)	8,360 (1.5%)	8	Cutaneous abscess, furuncle and carbuncle (L02)	4,178 (1.7%)	8	Other disorders of brain (G93)	4,784 (1.6%)
	9	Other disorders of brain (G93)	8,255 (1.5%)	9	Acute bronchiolitis (J21)	3,640 (1.5%)	9	Acute bronchiolitis (J21)	4,720 (1.6%)
	10	Cutaneous abscess, furuncle and carbuncle (L02)	7,794 (1.4%)	10	Other disorders of brain (G93)	3,471 (1.4%)	10	Cutaneous abscess, furuncle and carbuncle (L02)	3,616 (1.2%)
Children aged 5–17.99 years	*n* = 331,260			*n*=162,320			*n* = 168,940		
	1	COVID-19 (U07.1)	40,581 (12.3%)	1	COVID-19 (U07.1)	20,374 (12.6%)	1	COVID-19 (U07.1)	20,207 (12%)
	2	Pneumonia, unspecified organism (J18)	31,302 (9.4%)	2	Pneumonia, unspecified organism (J18)	15,335 (9.4%)	2	Pneumonia, unspecified organism (J18)	15,967 (9.5%)
	3	Trauma and injuries (summary code)	19,794 (6%)	3	Acute appendicitis (K35)	9,956 (6.1%)	3	Trauma and injuries (summary code)	15,059 (8.9%)
	4	Acute appendicitis (K35)	18,913 (5.7%)	4	Acute bronchitis (J20)	6,776 (4.2%)	4	Acute appendicitis (K35)	8,957 (5.3%)
	5	Acute bronchitis (J20)	14,051 (4.2%)	5	Chronic disease of tonsils and adenoids (J35)	6,158 (3.8%)	5	Acute bronchitis (J20)	7,275 (4.3%)
	6	Acute tonsillitis (J03)	11,865 (3.6%)	6	Acute tonsillitis (J03)	5,844 (3.6%)	6	Acute tonsillitis (J03)	6,021 (3.6%)
	7	Chronic disease of tonsils and adenoids (J35)	11,525 (3.5%)	7	Malaise and fatigue (R53)	4,806 (3%)	7	Chronic disease of tonsils and adenoids (J35)	5,367 (3.2%)
	8	Noninfective gastroenteritis and colitis (K52)	8,429 (2.5%)	8	Trauma and injuries (summary code)	4,735 (2.9%)	8	Noninfective gastroenteritis and colitis (K52)	4,276 (2.5%)
	9	Cutaneous abscess, furuncle and carbuncle (L02)	7,558 (2.3%)	9	Noninfective gastroenteritis and colitis (K52)	4,153 (2.6%)	9	Cutaneous abscess, furuncle and carbuncle (L02)	4,051 (2.4%)
	10	Malaise and fatigue (R53)	7,482 (2.3%)	10	Cutaneous abscess, furuncle and carbuncle (L02)	3,507 (2.2%)	10	Malaise and fatigue (R53)	2,676 (1.6%)

COVID-19, coronavirus disease 2019.

**Table 3:** The ten most frequent ICD-10-coded (13) main diagnoses and their crude hospital mortality rates, the ten most frequent ICD-10-coded (13) main diagnoses among hospitalizations ending in death, and the ten most frequent ICD-10-coded (13) main diagnoses with the highest crude hospital mortality rates in the three age groups.

Age groups	Most Frequent Main Diagnoses and Their Crude Hospital Mortality Rates *n* (%)			Main Diagnoses among Hospitalizations Ending in Death *n* (% of all deaths)			Main Diagnoses with the Highest Crude Hospital Mortality Rates *n* (%)		
Newborns (<28 days)	1	Neonatal jaundice (P59)	6/21,646 (0.02%)	1	Respiratory distress of the newborn (P22)	872 (30.7%)	1	Trisomy 18 and Trisomy 13 (summary code)	11/12 (91.7%)
	2	Disturbances of cerebral status of the newborn (P91)	539/6,427 (8.4%)	2	Disturbances of cerebral status of newborn (P91)	539 (20.0%)	2	Congenital malformations, not classified (summary code)	51/56 (91.1%)
	3	Infections specific to the perinatal period (P39)	163/1,917 (8.5%)	3	Infections specific to the perinatal period (P39)	163 (6.1%)	3	Intracranial laceration and hemorrhage due to birth injury (P10)	18/28 (64.3%)
	4	Respiratory distress of the newborn (P22)	872/1,538 (56.7%)	4	Congenital malformations of the heart and circulatory system (summary code)	115 (4.3%)	4	Congenital malformation syndromes affecting multiple systems (summary code)	28/48 (58.3%)
	5	Pneumonia, unspecified organism (J18)	9/1,512 (0.6%)	5	Congenital pneumonia (P23)	90 (3.3%)	5	Congenital syphilis (A50)	34/59 (57.6%)
	6	Other respiratory conditions originating in the perinatal period (P28)	21/1,384 (1.5%)	6	Disorders of the newborn related to short gestation and low birth weight (P07)	77 (2.9%)	6	Respiratory distress of the newborn (P22)	872/1,538 (56.7%)
	7	Congenital pneumonia (P23)	90/1,064 (8.6%)	7	Congenital malformations, not classified (summary code)	51 (1.9%)	7	Congenital hydrocephalus (Q03)	9/16 (56.3%)
	8	Omphalitis of newborn (P38)	0/864 (0%)	8	Congenital malformations of digestive system (summary code)	51 (1.9%)	8	Neonatal aspiration (P24)	48/87 (55.2%)
	9	Viral pneumonia (non-COVID-19) (J12)	8/753 (1.1%)	9	Neonatal aspiration (P24)	48 (1.8%)	9	Congenital malformations of the heart and circulatory system (summary code)	115/218 (52.8%)
	10	COVID-19 (U07.1)	1/676 (0.1%)	10	Bacterial sepsis of the newborn (P36)	46 (1.7%)	10	Birth trauma (summary code)	8/19 (42.1%)
Under-five children (28 days—4.99 years)	1	Pneumonia, unspecified organism (J18)	209/202,598 (0.1%)	1	Congenital malformations of the heart and circulatory system (summary code)	214 (15.8%)	1	Congenital malformations, not classified (summary code)	27/32 (84.4%)
	2	Noninfective gastroenteritis and colitis (K52)	33/61,441 (0.05%)	2	Pneumonia, unspecified organism (J18)	209 (15.4%)	2	Chronic respiratory disease originating in the perinatal period (P27)	10/15 (66.7%)
	3	Acute bronchitis (J20)	0/53,703 (0%)	3	Trauma and injuries (summary code)	72 (5.3%)	3	Congenital malformation syndromes affecting multiple systems (summary code)	19/32 (59.4%)
	4	COVID-19 (U07.1)	14/29,779 (0.05%)	4	Other sepsis (A41)	57 (4.2%)	4	Congenital viral diseases (P35)	15/28 (53.6%)
	5	Viral pneumonia (non-COVID-19) (J12)	20/28,921 (0.07%)	5	Disturbances of cerebral status of the newborn (P91)	54 (4.0%)	5	Nontraumatic subarachnoid hemorrhage (I60)	6/15 (40.0%)
	6	Acute tonsillitis (J03)	1/16,140 (0.01%)	6	Down syndrome (Q90)	38 (2.8%)	6	Other sepsis (A41)	57/176 (32.4%)
	7	Acute laryngitis and tracheitis (J04)	3/15,666 (0.02%)	7	Noninfective gastroenteritis and colitis (K52)	33 (2.4%)	7	Meningococcal infection (A39)	9/28 (32.1%)
	8	Acute bronchiolitis (J21)	0/8,360 (0%)	8	Encephalitis, myelitis and encephalomyelitis (G05)	27 (2.0%)	8	Congenital syphilis (A50)	4/15 (26.7%)
	9	Other disorders of brain (G93)	25/8,255 (0.3%)	9	Lymphoid leukemia (C91)	27 (2.0%)	9	Intracranial nontraumatic hemorrhage of newborn (P52)	18/70 (25.7%)
	10	Cutaneous abscess, furuncle and carbuncle (L02)	2/7,792 (0.03%)	10	Congenital malformations of digestive system (summary code)	27 (2.0%)	10	Respiratory distress of newborn (P22)	3/12 (25.0%)
Children aged 5–17.99 years	1	COVID-19 (U07.1)	7/40,581 (0.02%)	1	Trauma and injuries (summary code)	86 (18.9%)	1	Other sepsis (A41)	20/108 (18.5%)
	2	Pneumonia, unspecified organism (J18)	22/31,302 (0.07%)	2	Lymphoid leukemia (C91)	39 (8.6%)	2	Nontraumatic subarachnoid hemorrhage (I60)	3/18 (16.7%)
	3	Trauma and injuries (summary code)	86/19,794 (0.4%)	3	Cerebral palsy (G80)	32 (7.0%)	3	Inflammatory polyneuropathy (G61)	3/18 (16.7%)
	4	Acute appendicitis (K35)	1/18,913 (0.01%)	4	Myeloid leukemia (C92)	23 (5.1%)	4	Congenital malformation syndromes affecting multiple systems (summary code)	2/15 (13.3%)
	5	Acute bronchitis (J20)	0/14,051 (0%)	5	Pneumonia, unspecified organism (J18)	22 (4.9%)	5	Nontraumatic intracerebral hemorrhage (I61)	7/53 (13.2%)
	6	Acute tonsillitis (J03)	0/11,865 (0%)	6	Other sepsis (A41)	20 (4.5%)	6	Congenital hydrocephalus (Q03)	2/16 (12.5%)
	7	Chronic disease of the tonsils and adenoids (J35)	0/11,525 (0%)	7	Malignant neoplasm of brain (C71)	13 (2.9%)	7	Infectious mononucleosis (B27)	2/18 (11.1%)
	8	Noninfective gastroenteritis and colitis (K52)	3/8,429 (0.04%)	8	Encephalitis, myelitis and encephalomyelitis (G05)	11 (2.4%)	8	Myeloid leukemia (C92)	23/286 (8.0%)
	9	Cutaneous abscess, furuncle and carbuncle (L02)	0/7,558 (0%)	9	Nontraumatic intracerebral hemorrhage (I61)	7 (1.5%)	9	Malignant neoplasm of brain (C71)	13/197 (6.6%)
	10	Malaise and fatigue (R53)	1/7,482 (0.01%)	10	Benign neoplasm of brain and other parts of central nervous system (D33)	7 (1.5%)	10	Encephalitis, myelitis and encephalomyelitis (G05)	11/169 (6.5%)

COVID-19, coronavirus disease 2019.

## Discussion

This descriptive study of the National Mongolian Hospital Data Registry is the first systematic and nationwide analysis of pediatric hospital admissions in Mongolia. Over a five years study period, we recorded 927,223 pediatric admissions to Mongolian hospitals. In line with the country's male to female sex ratio at birth of 1.04 (16), a slight predominance of male pediatric hospital admissions (53.8%) was observed. The median prevalence of pediatric hospital admissions per 100,000 population (all ages) per year was 5,218 (IQR, 4,386–6,098). This prevalence is four times lower than the one recorded for Mongolian adults (≥18 years) in a previous analysis of the same register [20,242 (IQR, 19,412–20,778)] (7). While the prevalence of adult hospital admissions in Mongolia was relevantly higher compared to other countries (7), the prevalence of pediatric hospital admissions as observed in the present analysis is comparable to numbers reported from countries such as England (17). Assuming a population size of 56,550,000 in England in mid-2020 (18), the prevalence of pediatric (<15 years) hospital admissions per 100,000 population (all ages) in England was 4,350 in 2020 (17). A United States database analysis, on the other hand, estimated a lower annual prevalence of pediatric hospital admissions per 100,000 population (all ages) of 1,603 in the United States in 2019 [assuming a United States population size of 328.3 millions in 2019 (19)], but only evaluated pediatric admissions to general hospitals and freestanding children's hospitals (20).

One quarter and two thirds of pediatric hospital admissions in Mongolia occurred in newborns and post-neonatal children under five years, respectively. This observation is in line with data from England, where the prevalence of pediatric hospital admissions was similarly highest among children aged 0–4 years (17). The higher prevalence of hospital admissions in children under the age of five years likely reflects the vulnerability of newborns and young children to perinatal conditions and infectious diseases during their first years of life (17). Prematurity, genetic disorders and congenital malformations are all well-known to further increase the risk for pathologies requiring hospital admissions in this age range (21–24).

Similar to hospital admissions of Mongolian adults (7), we found that the prevalence of pediatric hospital admissions per 100,000 population (all ages) per year was highest in level 2 hospitals with 64.5–74.5% of children across all age groups being admitted to these hospitals. This result mirrors the fact that level 2 hospitals are the only hospitals (provincial hospitals) in rural Mongolia offering specialized pediatric services. In the capital city of Ulaanbaatar, level 2 hospitals (district and private hospitals) are usually also the first point of referral for children requiring hospital admissions, while tertiary pediatric facilities are reserved for complex cases following referral through lower-level facilities. This finding underlines the crucial role of provincial and district hospitals in providing hospital-based healthcare services for children in Mongolia. Furthermore, it is an important signal for the future direction of resource allocations to improve hospital-based services for Mongolian children.

The main diagnoses leading to pediatric hospital admissions in our analysis did not relevantly vary between males and females but substantially differed between the three age groups (Tables 2, 3). While neonatal jaundice, cerebral disorders and respiratory conditions including pulmonary infections predominated as the main diagnoses among newborns, the most common reasons for hospital admission in post-neonatal children were infections of the respiratory tract and lungs as well as the gastrointestinal system. In children aged 5–17.99 years, COVID-19, pneumonia, as well as trauma and injuries were the three most common cause of hospital admission. These results are in accordance with prior data from Mongolia (25, 26) and other countries (17). For example, a population-based, cross-sectional study in rural Mongolia found that lower respiratory tract infections and diarrhea were the most frequent reasons for hospitalization among children up to the age of three years (25). Similarly, an analysis of the pneumonia surveillance program in Ulaanbaatar reported a high hospitalization rate due to lower respiratory tract infections among children aged 2–59 months (26). The observation of the latter study that the incidence of pediatric lower respiratory tract infections was lowest in summer months could explain why the prevalence of hospital admissions in post-neonatal children under five years in our study was lower during summer than other seasons. Interestingly and in contrast to results from a prior study evaluating hospital admissions in Mongolian adults (7), which reported that the ICD-10 code of tubulo-interstitial nephritis was erroneously used by Mongolian physicians as a code for unspecified, low-acuity conditions (*e.g.*, fatigue syndrome), this code was not found among the most common main diagnoses in children.

The observation period of our study included the two years (2021 and 2022), during which the COVID-19 pandemic affected Mongolia (27). With minimal differences between males and females, COVID-19 was the fourth most common main diagnosis in post-neonatal children under five years and the most common cause of hospital admission in children aged 5–17.99 years. These results are comparable to studies from other Asian countries and Europe (28, 29), reporting that pediatric hospitalization rates for COVID-19 were highest in infants and children older than 10 years. Systematic reviews and cohort studies confirmed that young children and those aged 10–18 years represented substantial proportions of pediatric COVID-19 cases, with adolescents being at higher risk for severe disease than younger children (28–30).

A striking result of our study was the crude all-cause hospital mortality rates and most common causes of in-hospital deaths in Mongolian children. With 5.9%, newborns had the by far highest crude mortality of the three age groups. Mortality rates in post-neonatal children under five years and children aged 5–17.99 years were 0.25 and 0.14%, respectively. This is consistently lower than the hospital mortality rates reported for hospitalized Mongolian adults (0.66%) (7). Most hospital deaths in newborns occurred on the day of birth or during the first week of life. Similarly, the majority of hospitalized post-neonatal children under five years died in their first year of life. While the hospital mortality rates of post-neonatal children in our study are largely similar to or even lower than those reported from other countries such as the United States (31, 32), the neonatal mortality in our study was high compared to data from the United States or Europe (31, 33, 34). However, when interpreting neonatal mortality data in our cohort, it needs to be kept in mind that we did not assess gestational age, which is a key determinant of neonatal mortality (35).

Our results on causes of pediatric in-hospital deaths may inform future strategies to increase survival of hospitalized children in Mongolia. Importantly, most causes of death identified in our study can be considered preventable. Therefore, strategies to decrease the risk of both lower respiratory tract infections [*e.g.*, vaccinations, modification of known risk factors (25)] and injuries in Mongolian children seem to be crucial interventions to reduce in-hospital deaths. In support of this, a cross-sectional study investigating 3,390 deaths at sites with high child mortality rates in sub-Sahara Africa and Southern Asia suggested that interventions prioritizing antenatal, intrapartum, and postnatal care could have prevented most deaths among children younger than 5 years (36). The finding that respiratory distress and other lung pathologies were among the main diagnoses causing most in-hospital deaths in newborns and post-neonatal children under five years suggests that improved capacities for mechanical ventilation and neonatal/pediatric critical care services, particularly in level 2 hospitals, could be one way to decrease hospital mortality of newborns and infants in Mongolia (37). The result that congenital malformations of the heart and circulatory system caused most in-hospital deaths of post-neonatal children under five years implies that setting up a comprehensive pediatric heart surgery program, which does currently not exist in Mongolia, might be another approach to reduce in-hospital deaths of Mongolian children. Pediatric deaths from trauma and injuries can effectively be decreased by implementation of trauma networks (38). Similarly, centralization of pediatric oncology services or improvements of their capacities is likely to decrease pediatric deaths from malignancies such as leukemia.

The main strength of our study is that it analyzed data of all pediatric hospital admissions in Mongolia over a five years period with only very few missing data. Accordingly, the study results reliably reflect the situation of pediatric hospitalization in the country. It included a large sample size enhancing the precision of our estimates. Furthermore, study data were extracted from a systematically maintained registry, which contains clearly defined variables. On the other hand, important limitations need to be considered when interpreting the results of our analysis. First, the National Hospital Data registry has not been systematically or externally validated. This shortcoming may particularly affect the accuracy with which secondary outcomes could be reported. Second, we could only evaluate a rather limited number of study variables that did not allow for a more detailed data analysis. Third, in the present analysis, hospital admissions were counted per admission and not per unique patient. This may potentially have inflated the hospital admission rates due to repeated admissions or inter-hospital transfers. Finally, we did not report ethnicity data as these are not included in the database. Accordingly, our data do not allow insights into possible disparities in the prevalence, main diagnoses, and outcomes of pediatric hospital admissions in Mongolia.

In conclusion, this nationwide, registry-based study found a median prevalence of 5,218 pediatric hospital admissions per 100,000 population (all ages) per year in Mongolia. The majority of pediatric hospital admissions occurred in post-neonatal children under five years. The observation period included the COVID-19 pandemic years.

## Data Availability

The data that support the findings of this study are available from NM but restrictions apply to the availability of these data, which were used under licence for the current study, and so are not publicly available. Data are however available from the authors upon reasonable request and with permission of the National Statistics Office of Mongolia.
