# Supplementary material for: Prevalence, main diagnoses, and outcomes of hospital admissions in Mongolian children: a national registry-based descriptive analysis
Source: Front Pediatr. 2026 Feb 10;14:1732305. doi: 10.3389/fped.2026.1732305 (PMC12929383; doi:10.3389/fped.2026.1732305)

Electronic Supplementary Material Figure E1. Study Flow Diagram.

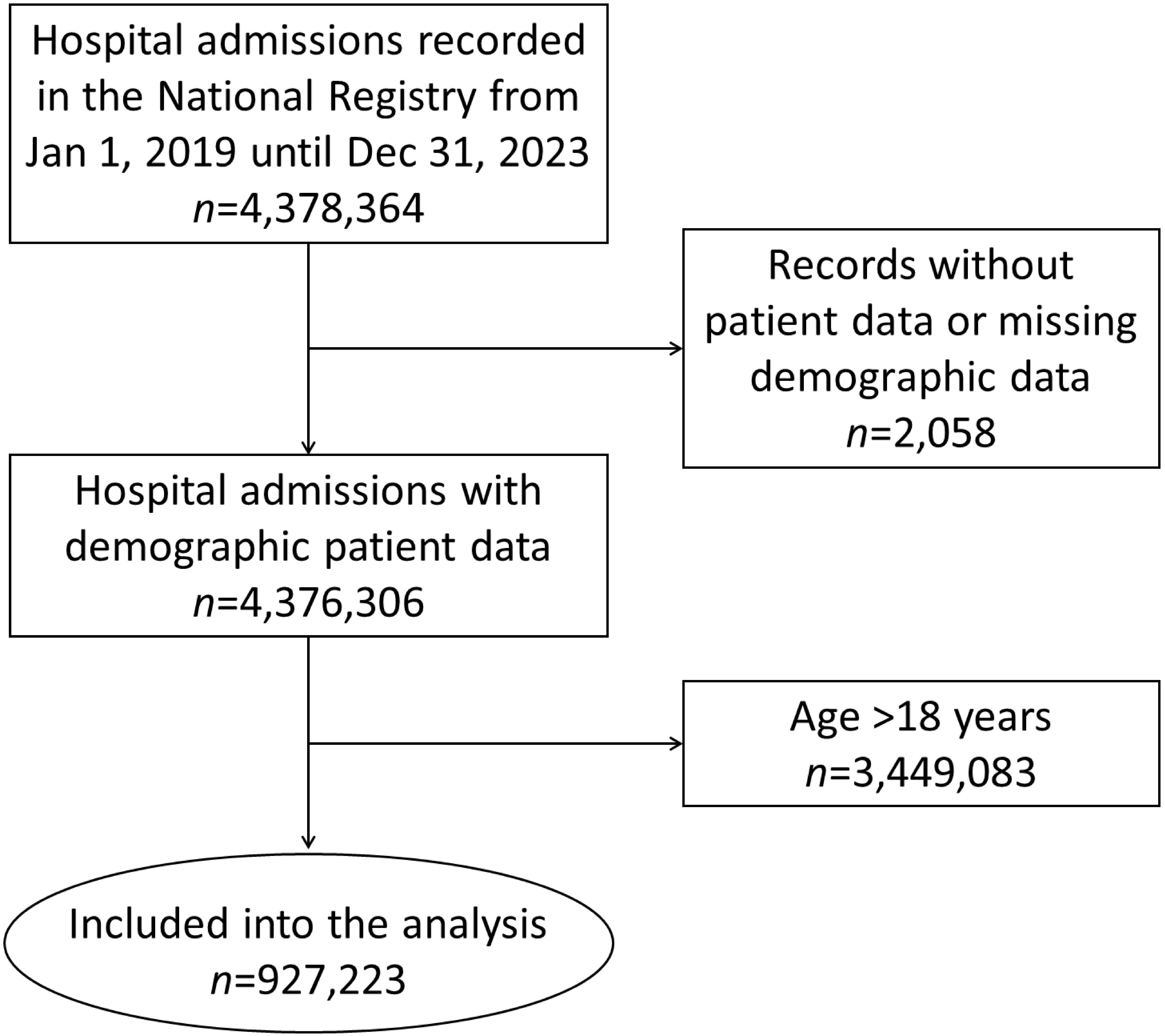

Electronic Supplementary Material Figure E2. Missingness maps of all study data stratified by age groups.

Newborns

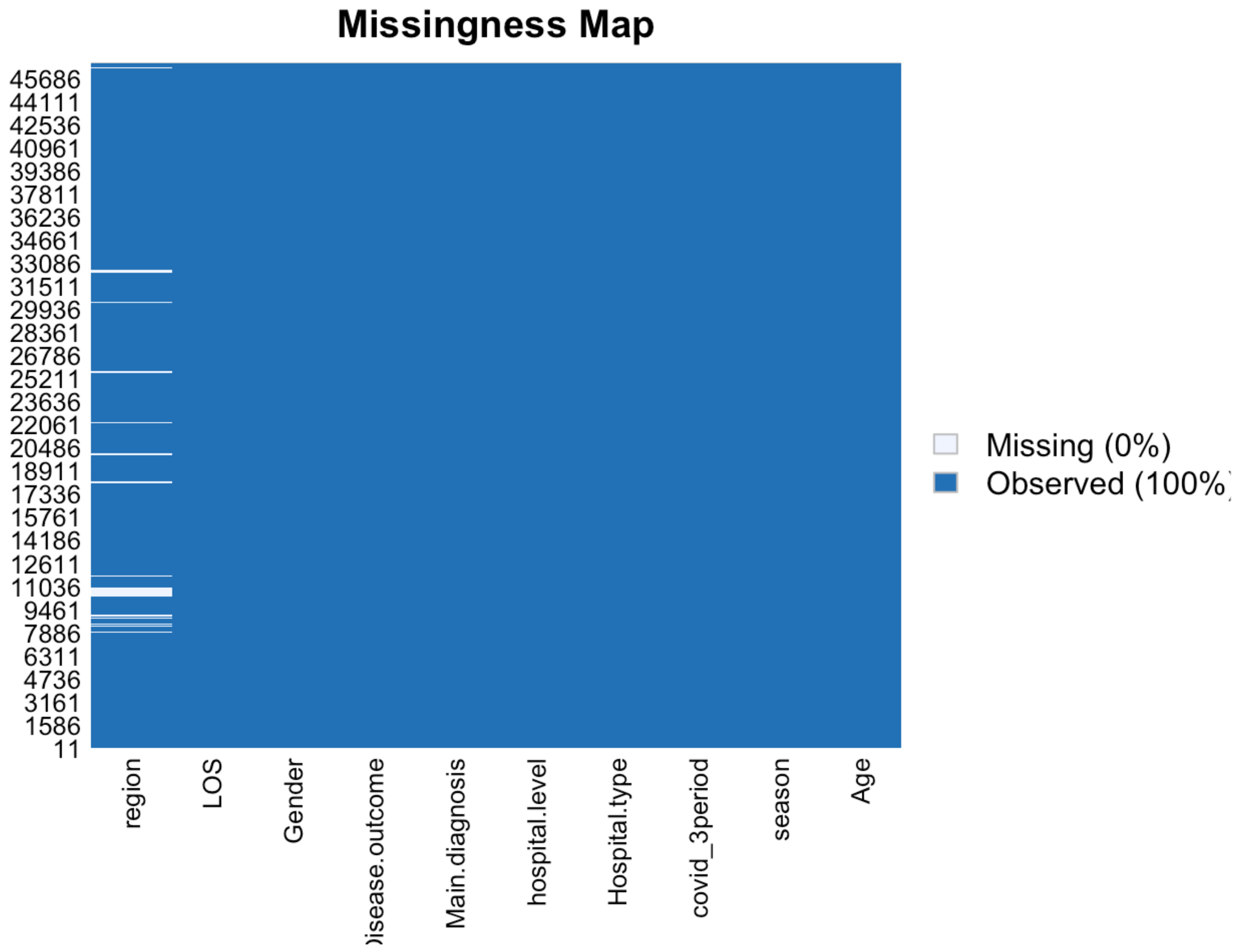

Under-five children

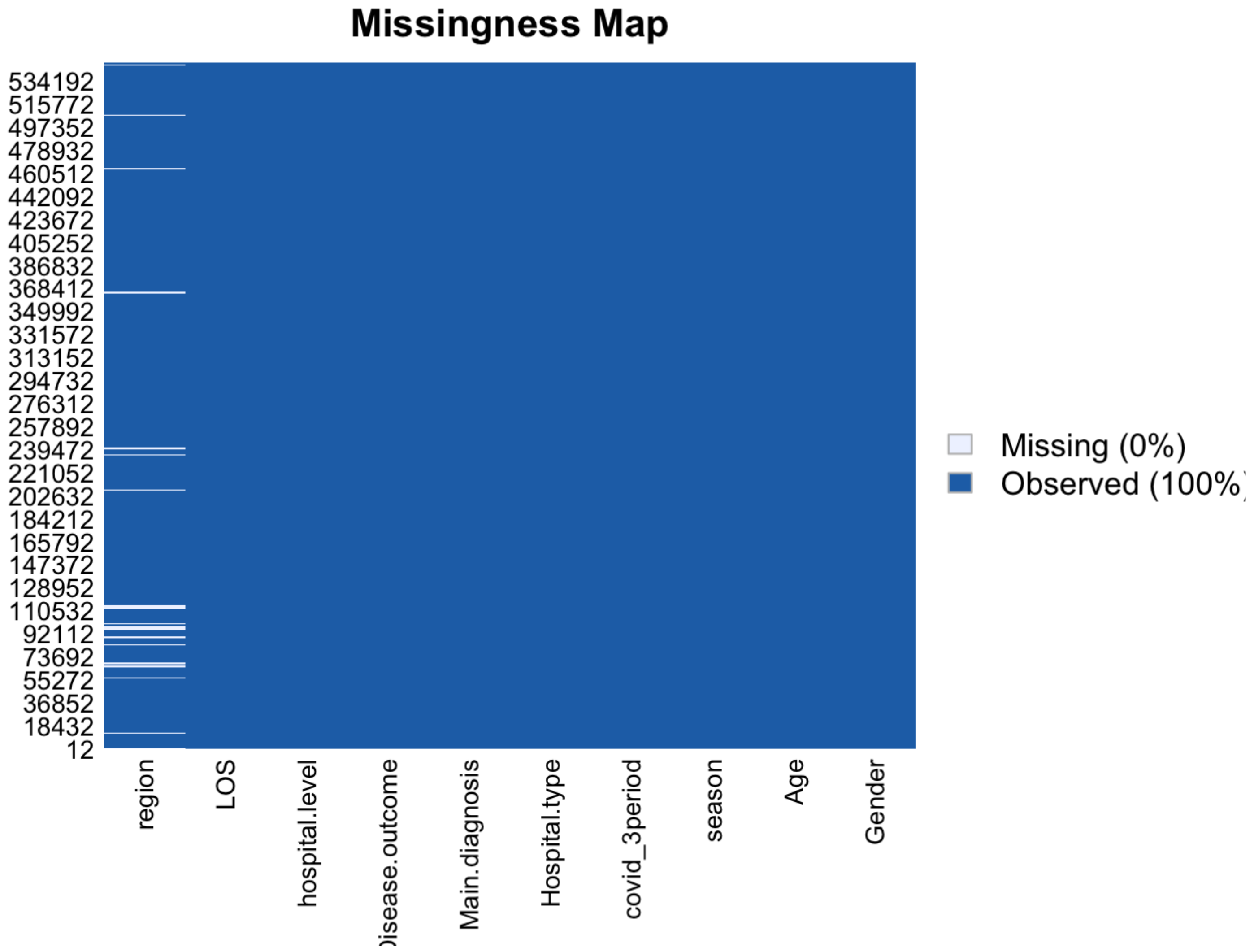

Children aged 5-17.99 years

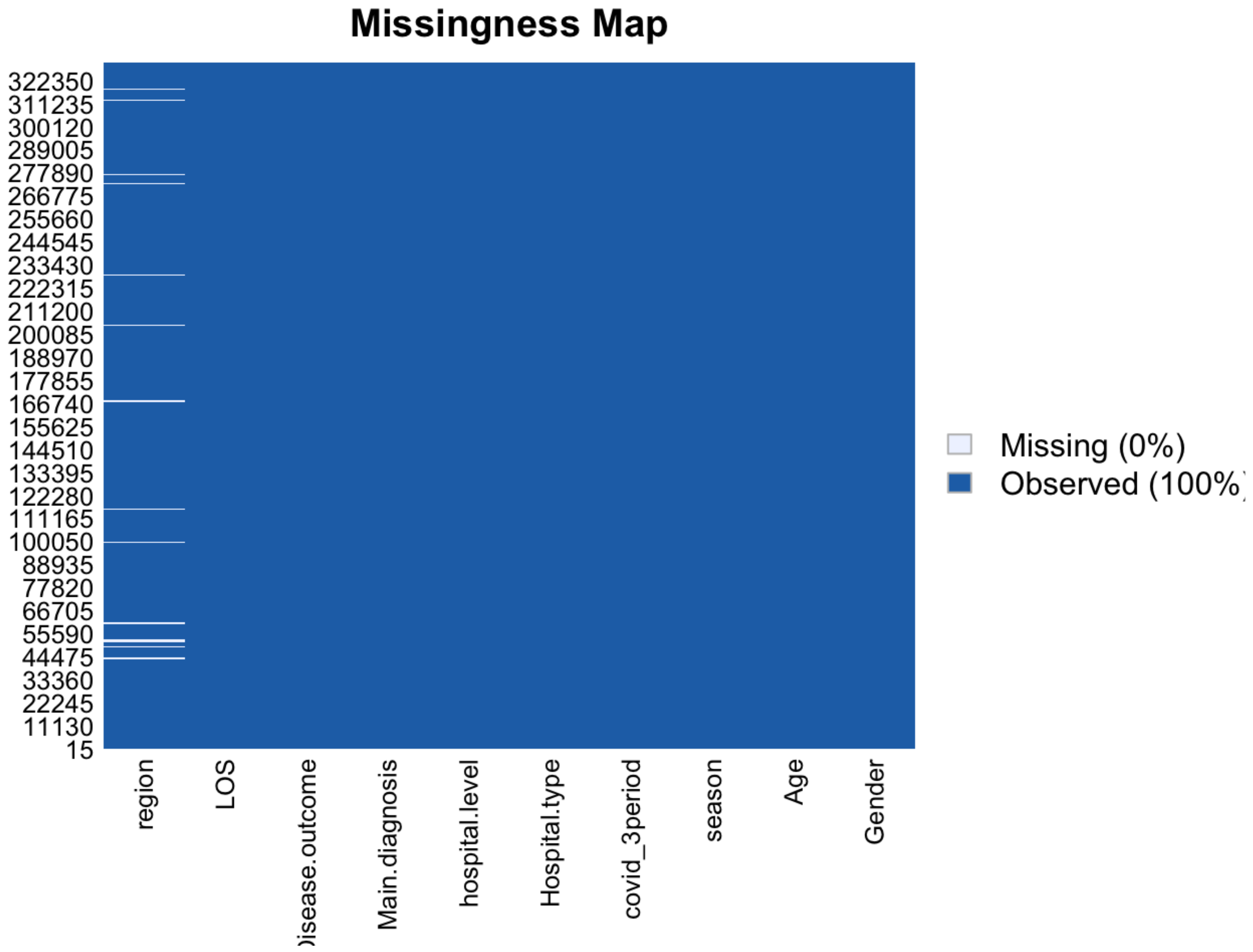

Supplement: Supplementary file 1 [file Datasheet1.pdf]
